# Trends of Sinusitis-Associated Orbital Cellulitis in Pediatric Patients: A Retrospective Cohort Review

**DOI:** 10.7759/cureus.84505

**Published:** 2025-05-20

**Authors:** Saleh Khurshied, Hira G Shah, Aisha Babar, Asma Afsar, Muhammad A Zahid, Mehrun Nisa

**Affiliations:** 1 Otolaryngology - Head and Neck Surgery, Pakistan Institute of Medical Sciences, Islamabad, PAK; 2 Ophthalmology, Alshifa Trust Eye Hospital, Rawalpindi, PAK; 3 Otolaryngology - Head and Neck Surgery, University Hospitals Plymouth NHS Trust, Plymouth, GBR; 4 Ophthalmology, Monash Health, Clayton, AUS; 5 Medicine and Surgery, Pakistan Institute of Medical Sciences, Islamabad, PAK

**Keywords:** clinical findings, common factors, orbital celluliits, sinusitis, trends analysis

## Abstract

Background and objective

Orbital cellulitis is mostly associated with sinusitis. The pediatric cases have a peculiar clinical presentation and variability with respect to different factors like season, type of sinus involved, and number of sinuses involved. In this study, we aimed to analyze the trends of sinusitis-associated orbital cellulitis in pediatric patients, which, we believe, would help physicians in managing these patients.

Material and methods

We retrospectively reviewed the discharge records of 74 pediatric patients treated at the ENT-Head and Neck Surgery Department of the Pakistan Institute of Medical Sciences, Islamabad, Pakistan, between January 2015 and March 2025. The study included only inpatients aged 1-14 years who were diagnosed with orbital cellulitis secondary to associated sinusitis, confirmed by CT findings. Data on patients’ demographics, clinical characteristics, month of presentation, type of sinusitis, and sinuses involved were recorded. Trends in the form of results were presented in tables and figures.

Results

The mean age of the cohort was 7 ± 4.3 years, with 47 (63.51%) being male and 27 (36.49%) female. Out of all the cases admitted, 70 (94.6%) had fever at the time of presentation, 74 (100%) had headache, 74 (100%) had pain, and 74 (100%) had chemosis and periorbital edema. Of the patients, 41 (55.40%) had ophthalmoplegia, 29 (39.19%) had facial edema, and 14 (18.92%) had reduced eyesight. There were 49 (66.22%) known cases of chronic sinusitis. Of the total number of cases, 29 (39.19%) occurred in the winter, 18 (24.32%) in the spring, 17 (22.97%) in the summer, and 10 (13.51%) in the fall. Of the total cases, 23 (31.08%) involved a single sinus, 46 (62.16%) involved two or more sinuses, and five (6.76%) involved pansinusitis. In cases with single sinus sinusitis, the ethmoid was most frequently involved (72.4%).

Conclusions

We observed that cases of sinusitis-associated orbital cellulitis had a peculiar presentation including headache, discomfort, chemosis, periorbital pain, and fever. Orbital cellulitis was more frequently linked to chronic rhinosinusitis. Orbital cellulitis linked to sinusitis peaked in the winter and spring. The most frequent cause of orbital cellulitis in children was ethmoidal sinusitis. Orbital cellulitis was typically caused by sinusitis involving two or more sinuses.

## Introduction

Acute post-septal orbital infections, such as orbital cellulitis, are typically caused by bacteria; their serious side effects could include subdural empyema, meningitis, cavernous sinus thrombosis, blindness, and even brain abscess [1]. Conjunctival chemosis, ophthalmoplegia, pain with ocular movements, and proptosis are clinical manifestations that can be relied upon to diagnose this illness. MRI and orbital CT scans help in establishing the diagnosis. Although it affects people of all ages, it is more commonly seen in children [1]. The symptoms of orbital cellulitis appear suddenly and worsen quickly. Early diagnosis and prompt treatment of the condition can help prevent complications and difficulties associated with it [1].

Ethmoid sinus involvement in younger individuals is the main culprit in the involvement ocular orbit region [2-6]. Weak ethmoid-orbital wall, particularly in youngsters, and the tight anatomical relationships between the eye orbit cavity and the ethmoid sinuses are the primary causes of this [7,8]. About 74-80% of the complications from acute sinusitis are orbital. While all age groups are susceptible, pediatric populations are more likely to experience it [9].

The orbit is a closed, sterile compartment with an orbital septum in front of it and bony walls on all sides. Orbital infections can present with a wide range of symptoms caused by inflammation of the orbit and periorbital tissues as a result of infectious disease [10]. The lamina papyracea forms a lateral barrier to the osteomeatal complex, which is where the drainage of the maxillary, ethmoidal, and frontal sinuses into the nasal cavity converges [11]. When inflammation brought on by viral or allergic rhinitis blocks this complex, it disrupts mucociliary clearance, alters local pH and oxygen and carbon dioxide concentrations, and sets the stage for bacterial sinusitis later on [12]. While bacterial sinusitis is most commonly associated with the fall and winter, it can occur at any time of the year [13].

Bacterial sinus infections can cause orbital cellulitis if they are not promptly or effectively treated. They can spread to the orbit through anatomizing, valveless veins or by direct extension through the paper-thin lamina papyracea. A dangerous ophthalmologic condition, orbital cellulitis can spread quickly and cause catastrophic side effects, such as subperiosteal abscess, cavernous sinus thrombosis, cerebral abscesses, permanent vision loss, and even mortality [14,15]. Trauma, surgical causes, blood-borne and dental infections, or the nearby extension of pathogenic agents from the sinuses, nasopharynx, and adjacent areas can all result in orbital infections [16,17]. The incidence of orbital cellulitis resulting from bacterial sinusitis is poorly understood, even though the prevalence of this illness is known to vary with the seasons [18].

In the emergency room, children's eyelid swelling and/or redness are not uncommon [19]. To preserve vision and avoid life-threatening complications, it is critical to identify the underlying orbital complications. Early management and the introduction of antimicrobial therapy are essential in these cases [20,21]. A multidisciplinary approach under the supervision of pediatricians, otolaryngologists, and ophthalmologists has been recommended to enhance diagnosis and clinical results, particularly in severe cases [22-25]. This study aims to evaluate the trends of sinusitis-associated orbital cellulitis in pediatric patients. We believe that our findings will help in the early recognition of the disease and facilitate better management of the patients, thereby optimizing patient outcomes and preventing complications.

## Materials and methods

This retrospective cohort review involved all pediatric patients with sinusitis-associated orbital cellulitis admitted to the ENT-Head and Neck Surgery inpatient department at the Pakistan Institute of Medical Sciences, Islamabad, Pakistan, over a period of 10 years between January 2015 and March 2025. We obtained formal written consent from the departmental head for using departmental data for conducting this research. To preserve patient confidentiality, information was collected from the discharge form of each patient, and any information that could help in the identification of the patient was hidden while the data were being collected. None of the patients or their family members were contacted directly.

This study only included pediatric patients between the ages of 1 and 14 years who had been diagnosed with orbital cellulitis due to associated sinusitis, as confirmed by CT findings. Patients having radiological evidence of sinusitis (single or more than one) in any sinus, like maxillary, frontal, ethmoid, and/or sphenoid regions in conjunction with orbital cellulitis, were considered to have concurrent sinusitis. The following factors were evaluated: age, gender, clinical presentation, month of presentation, type of sinusitis, and sinuses involved. Other orbital infections and cases of orbital cellulitis from other causes were not included. Adult population and patients with any other associated sinusitis complications were also excluded

SPSS Statistics version 25.0 (IBM Corp., Armonk, NY) was employed to statistically evaluate the collected data. For each variable, numbers and percentages were computed. Descriptive data were presented as frequencies and percentages, and tables and charts were used to visualize the data.

## Results

**Figure 1:**
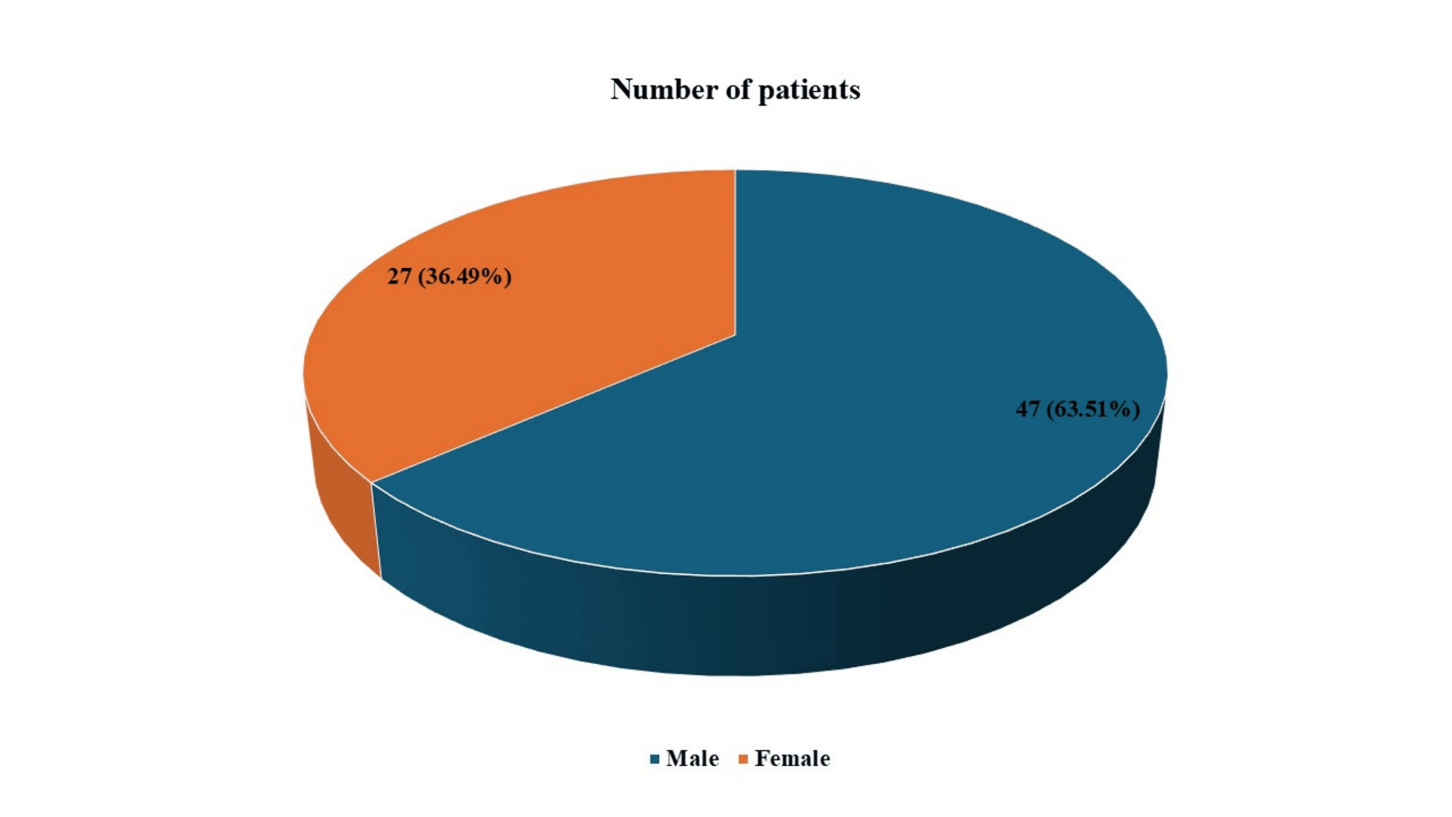
Trends with respect to gender of patients with sinusitis-associated orbital cellulitis

The mean age of the cohort was 7 ± 4.3 years; there were 47 (63.51%) males and 27 (36.49%) females, as presented in Figure 1.

**Figure 2:**
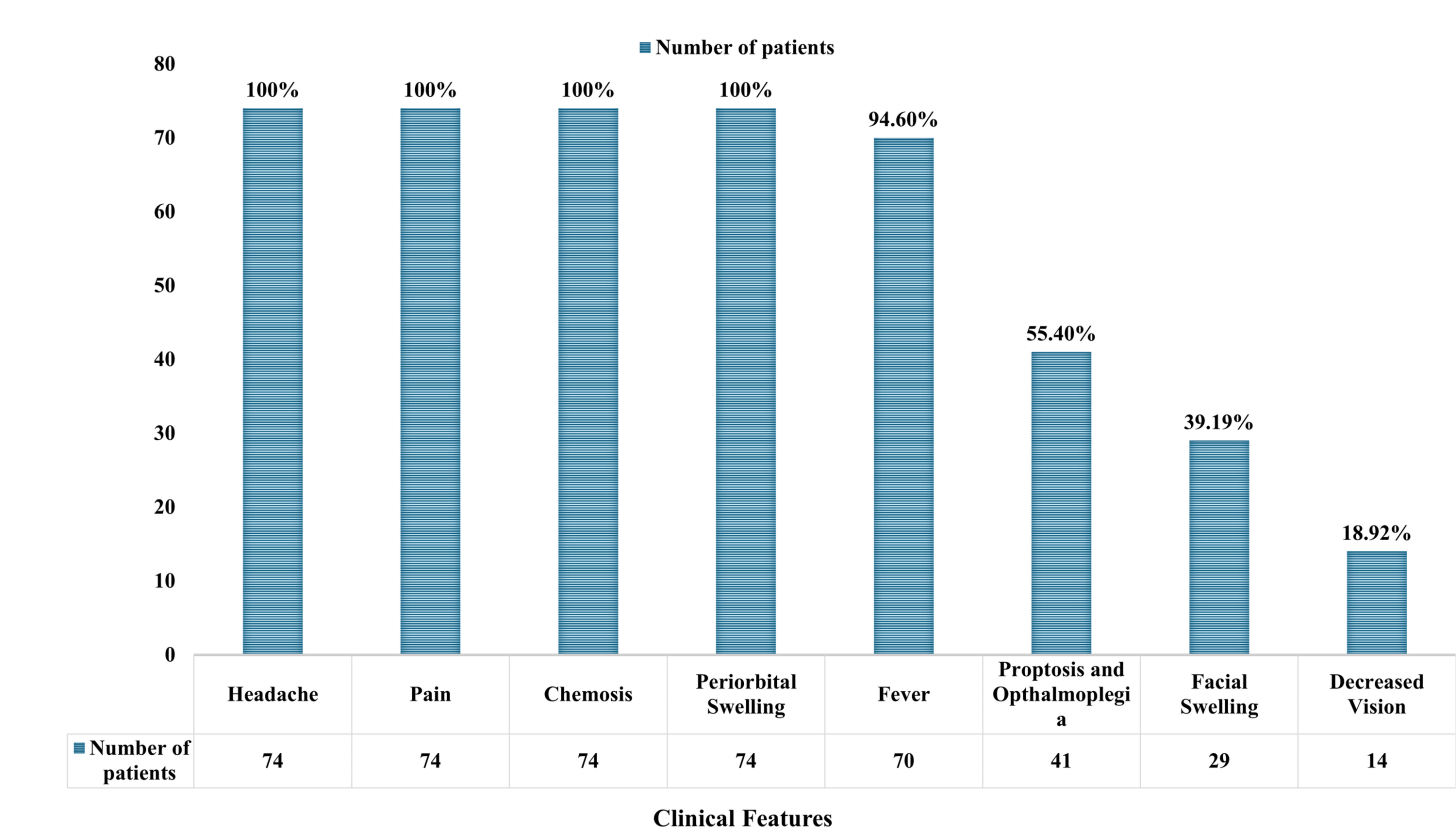
Clinical presentation of cases with sinusitis-associated orbital cellulitis

At the time of presentation, all 74 (100%) cases had headache, 74 (100%) had pain, and 74 (100%) had chemosis and periorbital edema. Of note, 70 (94.6%) had fever, 41 (55.40%) had ophthalmoplegia, 29 (39.19%) had facial edema, and 14 (18.92%) had reduced eyesight. These trends are plotted in Figure 2.

**Figure 3:**
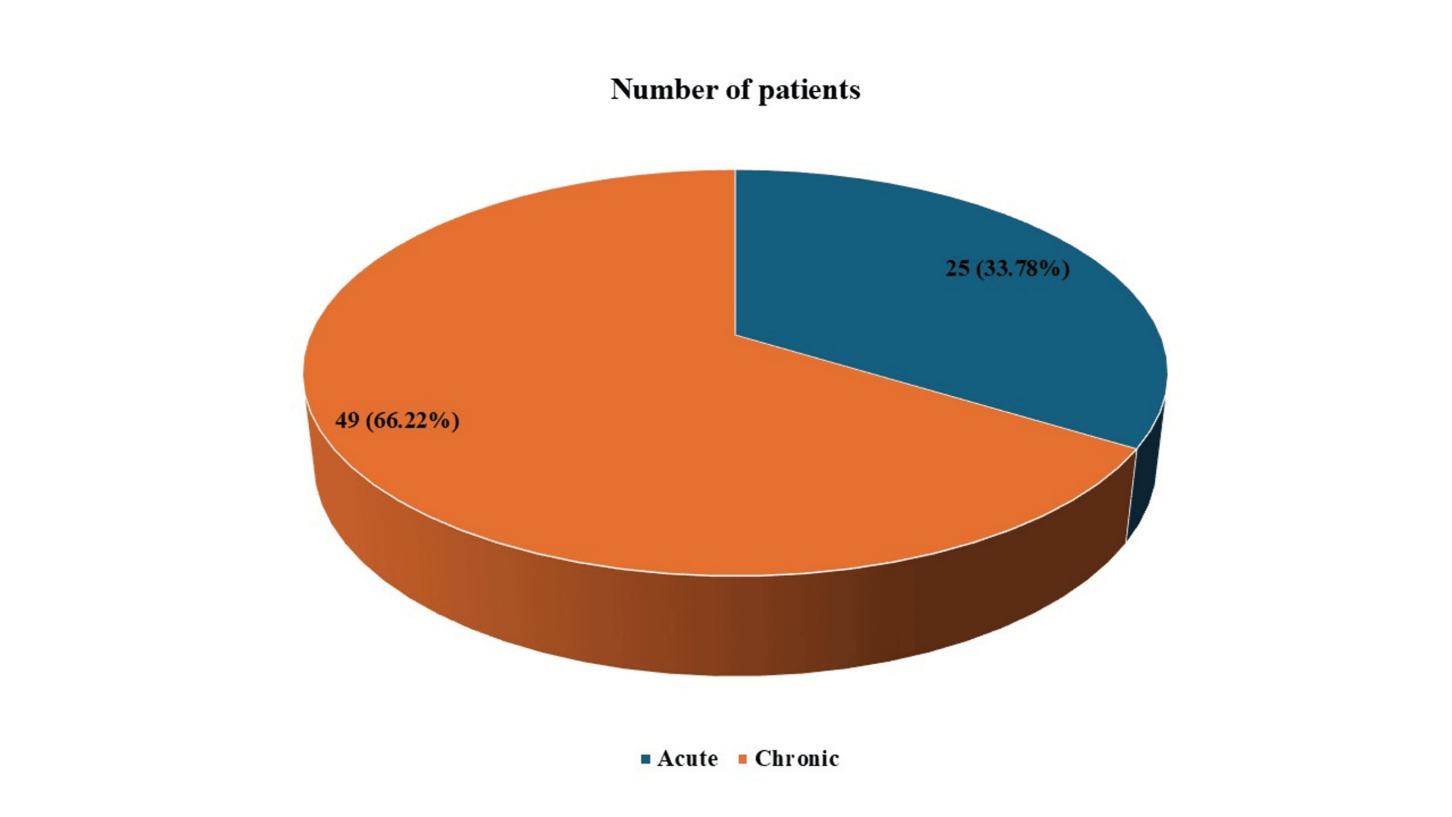
Type of sinusitis in patients with sinusitis-associated orbital cellulitis

As shown in Figure 3, there were 25 (33.78%) cases of acute sinusitis and 49 (66.22%) known cases of chronic sinusitis.

**Figure 4:**
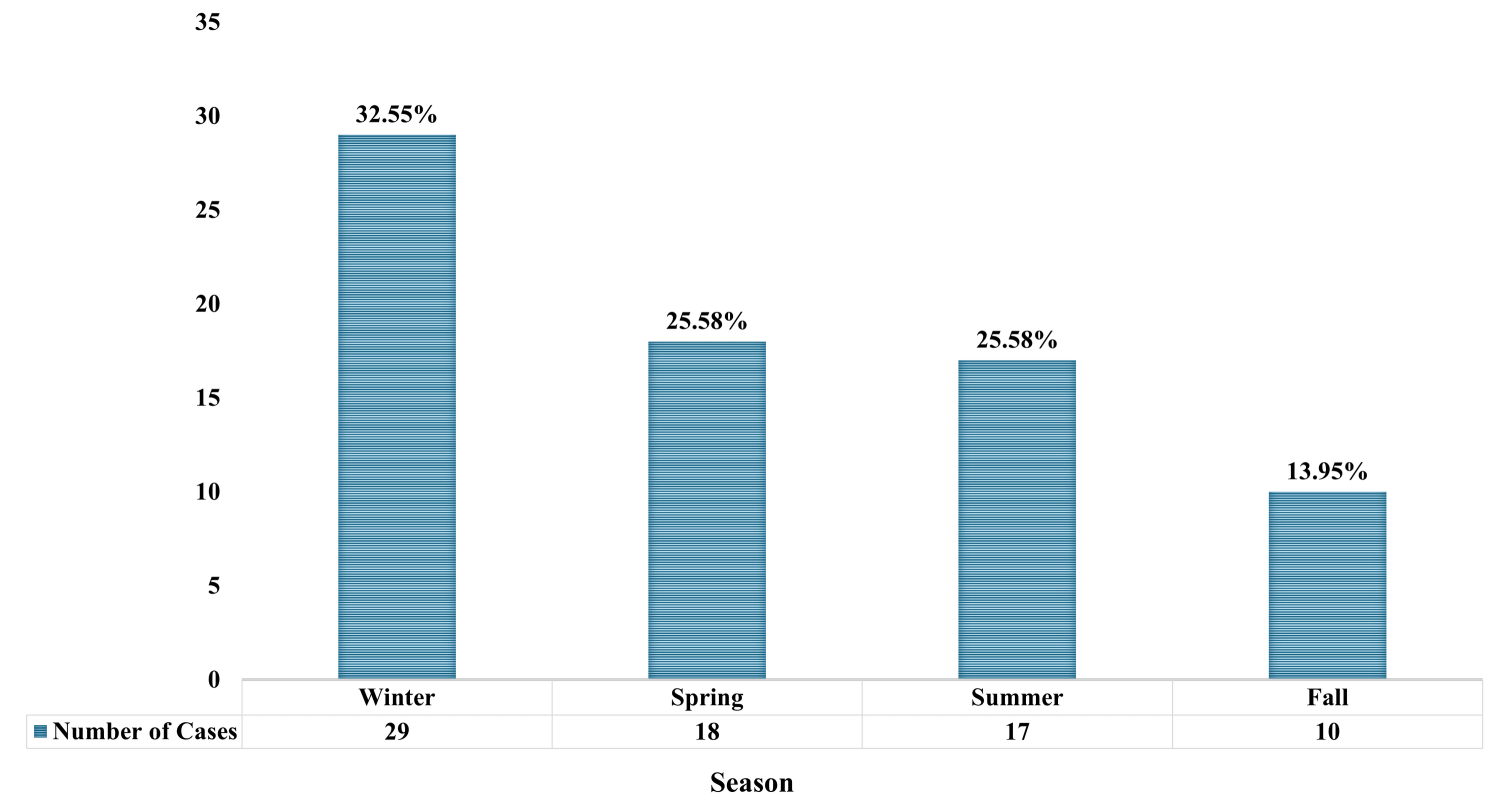
Trends of sinusitis-associated orbital cellulitis in different seasons

Of the total number of cases, 29 (39.19%) occurred in the winter, 18 (24.32%) in the spring, 17 (22.97%) in the summer, and 10 (13.51%) in the fall, as presented in Figure 4.

**Figure 5:**
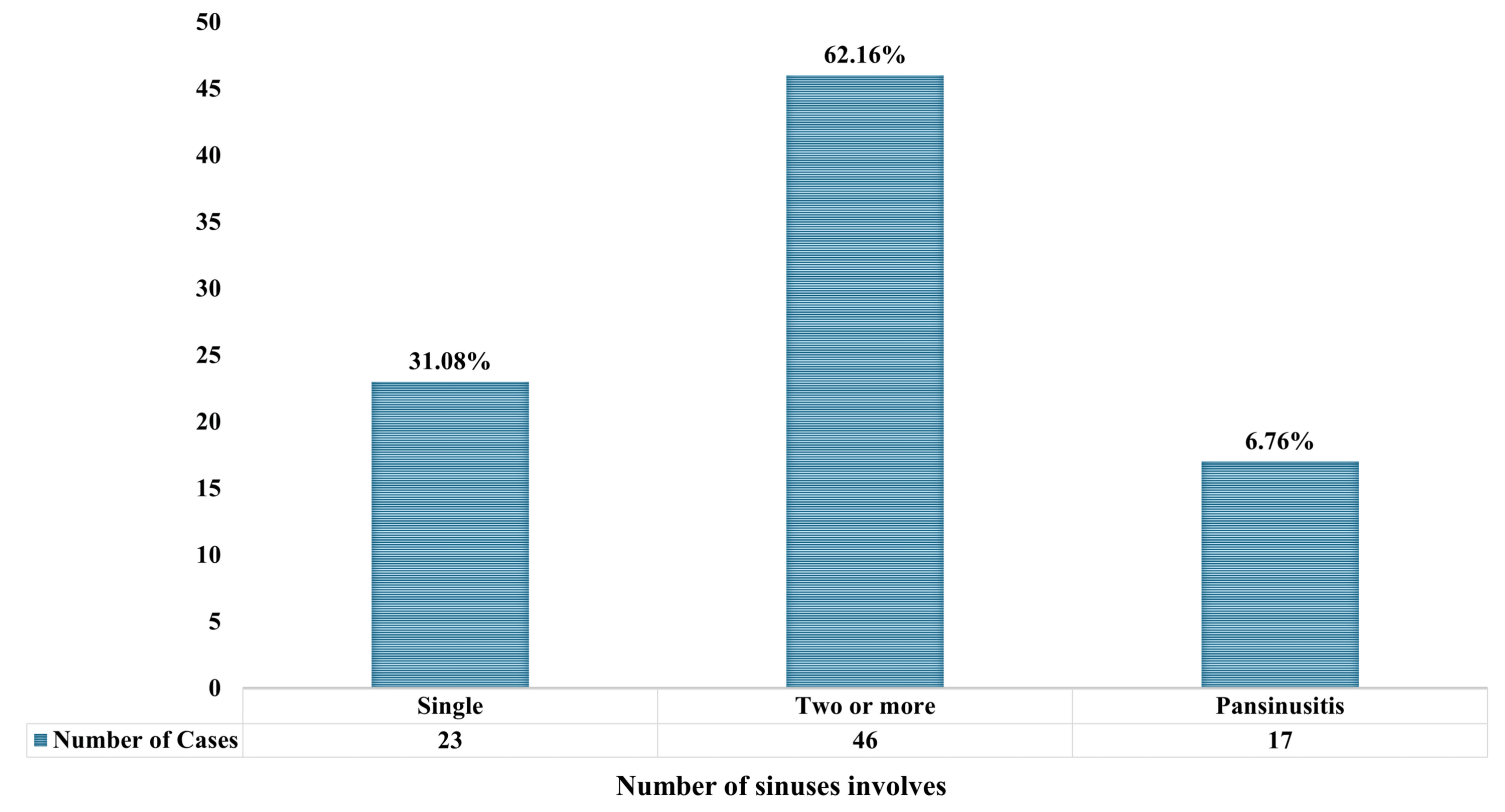
Number of sinuses involved in sinus-associated orbital cellulitis

Of the total cases, 23 (31.08%) involved a single sinus, 46 (62.16%) involved two or more sinuses, and five (6.76%) involved pansinusitis, as shown in Figure 5. In cases of single sinus sinusitis, the ethmoid was most frequently involved (21/29, 72.4%), while in cases of two sinus sinusitis, the ethmoid and maxillary were most frequently involved (36/46, 78.26%).

## Discussion

We discussed the trends related to various features and associations of orbital-associated sinusitis in the pediatric population. In their study of sinusitis-related orbital cellulitis, Mekhitarian Neto et al. [26] observed that the majority of patients were men, with a median age of 6.5 years. The most commonly affected sinus was the maxillary sinus. While our results were similar to these, we had a higher prevalence of ethmoid sinusitis. Males predominated in both the pediatric and adult groups of the study by Raman et al. [27]. Children were the most commonly impacted age group, followed by people in their 30s and 40s, in Pandian et al.'s study [28]. Wong et al. [9] reported similar findings.

According to research by Khurshied et al. [29], sinusitis is the most common cause of orbital infections. There is proven seasonal variation in both infectious sinusitis and seasonal allergic rhinitis. While infectious sinusitis tends to occur in the fall and winter, allergic rhinitis is more prevalent in the spring [30]. Our findings were similar to those of Lee et al. [18], who reported that the sinus-related orbital cellulitis case burden decreased from June to September. Studies have shown that orbital cellulitis peaks in late fall to early spring [31,32], but there was no discernible seasonal correlation. Similar to our findings, Kais et al. [33] demonstrated that there is a seasonal susceptibility for orbital problems, primarily in the winter and spring seasons.

According to Mathew et al. [34], children with ethmoidal sinusitis have a higher risk of orbital cellulitis. As per Ferguson and McNab [32] and Chaudhry et al., up to 91% of pediatric patients with orbital cellulitis also had sinus illness that was confirmed by radiography simultaneously. The most prevalent sinus implicated in children, according to a study by Al-Madani et al. [35], was the ethmoidal sinus. Additionally, nearly all of the patients in their study presented between November and February, which is consistent with our findings. Likewise, Fanella et al. [36] observed that the most commonly implicated sinus in their cases was the ethmoid. No research in the literature has clearly described the trends of clinical presentations of orbital cellulitis in the pediatric population. Orbital cellulitis is associated with features of lid swelling, headache, painful eye movement, diplopia, reduced visual acuity, ophthalmoplegia, and loss of light reflex in the literature [37]. Some similar trends were found in our study as well.

Our research identifies and sheds light on the trends of sinusitis-associated orbital cellulitis in pediatric patients, which will help promote the better management of patients and help physicians in decision-making. The strength of the study involves its long duration of over 10 years and the inclusion of a specified age group and type of orbital cellulitis. However, the study has certain limitations, primarily the small sample size and a single-center design. Moreover, comorbidities and etiological types of rhinosinusitis were not documented. The retrospective design and the rarity of the condition we explored also further limit the generalizability of our findings.

## Conclusions

Our findings showed that all cases had a peculiar presentation of sinusitis-related orbital cellulitis, such as headache, discomfort, chemosis, periorbital pain, and fever. Orbital cellulitis was more frequently linked to chronic rhinosinusitis. Orbital cellulitis linked to sinusitis peaked in the winter and spring. The most common cause of orbital cellulitis in children was ethmoidal sinusitis. Orbital cellulitis was typically caused by sinusitis involving two or more sinuses.
